# Institutional presence: Toward a further developed Community of Inquiry model integrating institutional functions in online and blended learning environment

**DOI:** 10.3389/fpsyg.2023.1132204

**Published:** 2023-03-13

**Authors:** Wei Zhang, Chang Zhu

**Affiliations:** ^1^Higher Education Institute, Beijing University of Technology, Beijing, China; ^2^Higher Education Institute, Vrije Universiteit Brussel, Brussels, Belgium

**Keywords:** institutional presence, Community of Inquiry, model, blended learning, online learning

## Abstract

In this research we examine the Community of Inquiry model and further develop the model by integrating a complementary institutional presence. For this purpose, a questionnaire including five presences and 73 questions was designed. In total, a response of 762 questionnaires from five universities were collected. Correspondingly, statistical analysis like factor analysis and structural equation model were conducted. The present paper is, duly, a quantitative investigation of the correlations between institutional presence and other presences in the new model as well. Finally, a further developed Community of Inquiry model that integrates institutional presence is generated. With a relatively large sample, the results meet the applicable requirements, indicating that the generated model is acceptable and fits well with the data.

## 1. Introduction

In its simplest sense, blended learning refers to a combination of face-to-face and online learning. Despite the extensive research on online and blended learning conducted over the last decade, the development of theoretical models specific to these environments remains inadequate. Be that as it may, one of the most intriguing models that is worth pointing at as appealing enough is the Community of Inquiry (CoI) model developed by Garrison et al. (2000). Shea and Bidjerano (2010) then developed the CoI model by incorporating a learner presence. Albeit the CoI model provides a framework for the entire process of online or blended learning, some studies which tried to probe the implementation of CoI model also found that some institutional functions did hardly match the model well enough. Therefore, the authors believe that the model could be further developed and made more systematic by integrating an institutional presence to account for the institution’s functions in the learning process. Added to this is Crossan et al. (1999) mentioned that to systematically develop learning, personal and group learning should be institutionalized, while Parker’s (2008) assertion of the vital integrity of teaching and learning processes within institutions is worthwhile. Thus, the objective of this paper is to further develop the CoI model by incorporating a complementary institutional presence that accounts for institutional functions in the learning process. In doing so, it is hoped that CoI model could be optimized and preferably guide the online or blended learning process.

By virtue of the core rationale of the present paper, which lies in an accomplishment of a markedly systematic and constructively developed CoI model incorporating the entirety of the features that renders it a Further Developed Community of Inquiry (FDCoI) model, a further relevantly underpinning theoretical framework has been intently formulated in the literature review section, whilst the utterly directing research questions, hypotheses, participants as well as instruments are being illustrated in the methodology section. By the same token, descriptive statistics, a normal test, exploratory factor analysis, confirmative factor analysis and a structural equation model were intricately performed and comprehensively elucidated in the findings section. As a grounded theory, then, the further developed CoI model is subsequently generated in the discussion section, without a minimum indifference and disregard as far as this paper broad limitations, which the conclusion highlights, are concerned.

The primary objective of this research is to examine the CoI model (Garrison et al., 2000) and the revised CoI model that adds learner presence (Shea and Bidjerano, 2010) and to further develop these models by incorporating a complementary institutional presence. The study seeks to address the following specific research questions:

(1) Can institutional presence be integrated in the CoI model?
  The classical CoI model has three presences (dimensions): teaching presence, social presence, and cognitive presence. Shea and Bidjerano (2010) has developed the model by adding a fourth dimension: learner presence. In this paper, we attempts to add a fifth dimension: institutional presence. To achieve this purpose and answer this research question, a questionnaire with five presences is generated and quantitatively analyzed by statistical methods like factor analysis and structural equation model to check whether the new model with five presences fits well with the data or not.
(2) If so, how should institutional presence be integrated in the CoI model?
  If the newly proposed five-presence model is deemed appropriate, an additional line of inquiry that warrants attention is how to incorporate institutional presence into the CoI model in a manner that accurately reflects its structural relationship with other CoI presences. Specifically, the investigation seeks to ascertain whether institutional presence exhibits moderate to strong standardized loadings and significant interactions with the other four presences by analyzing the results obtained from the structural equation model.

The hypothesis of this study is that the CoI model could be further developed by the addition of an institutional presence. This development is based on a model for institution employment of online and blended learning in universities (Graham, et al., 2013). On the grounds of the CoI model and prior research, we contend that institutional presence exerts a substantial influence on cognitive presence and posits that it interacts with teaching, social and learner presence. To test this hypothesis and construct a novel model, descriptive statistics, a normal test, and exploratory factor analysis were conducted using SPSS21.0, followed by confirmative factor analysis and structural equation model using AMOS21.0.

## 2. Literature review

### 2.1. Community of Inquiry

**Figure 1:**
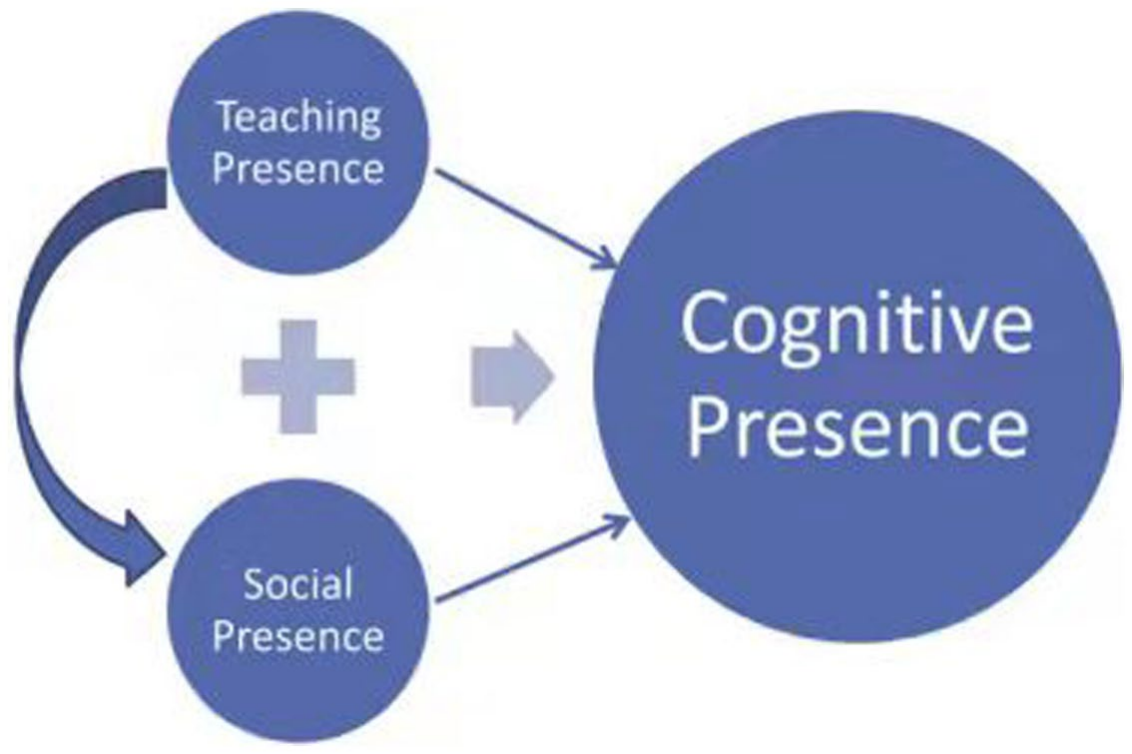
Relationships between teaching, social, and cognitive presence.

Despite the plethora of available online and blended learning models, research on the quality of online and blended learning has placed considerable emphasis on the CoI model, first introduced by Garrison et al. (2000, 2010). The CoI model comprises three dimensions: cognitive presence, social presence, and teaching presence. A significant amount of research has been conducted on the interrelations among these presences (Garrison et al., 2010), with most results indicating substantial effects among the three presences. Notably, a considerable number of findings substantially revealed the existence of a noteworthy impact among teaching presence, cognitive presence and social presence, of which the Figure 1 below is a concise outline of the crucial linkage amongst these three presences. In essence, teaching presence exerts a substantial effect on social presence, and both teaching and social presences have a notable impact on cognitive presence.

In its notable peculiarities, cognitive presence is associated with the student’s construction and confirmation of meaning, pertaining to course content, on the basis of sustained reflection and discourse within the CoI. Consequently, it has been a subject of research to a large extent by various scholars (Shea and Bidjerano, 2009), most of which asserted the that teaching and social presences significantly influence cognitive presence. Furthermore, recent studies have suggested that cognitive presence may be more explicitly demonstrated in deeper learning assignments beyond threaded discussions and chats, as asserted by Shea and Bidjerano (2009).

In terms of social presence, it refers to the learners’ capacity to present themselves as genuine individuals, both socially and emotionally, within the CoI. This area has been the subject of extensive research (Turk et al., 2022), with results emphasizing the degree to which video communication is likely to exert larger impact on social presence. Consequently, social presence has received the most attention among the three presences and has been notably linked with learning outcomes and learner satisfaction (Garrison and Arbaugh, 2007).

What ought to be inferred, accordingly, is that Teaching presence focuses on the organization, design and facilitation of the cognitive and social components of a course with the aim of achieving the sought-after educational outcomes. Additionally, a further noteworthy finding of research on social presence in the CoI model, which has expanded the realm of its exploration (Zhang et al., 2022), pertains to revealing the extent to which teaching presence is perceived in diverse groups. Moreover, a great deal of evidence suggests that teaching presence is closely associated with student satisfaction, perceived learning and a sense of community (Shea et al., 2006).

Despite the extensive body of studies have supported the CoI as a model of online and blended learning, further development of best practices that promote an educational community is warranted. For instance, Garrison and Arbaugh (2007) have suggested that further research should evaluate all three presences simultaneously using improved methodologies while advocating for the joint reconstruction of these concepts. Previous research has focused on one of the presences ignoring its interconnections with the other presences, and has often emphasized online learning more generally rather than specific disciplines. Nevertheless, Garrison and Arbaugh strongly recommend research that examines the implementation of the CoI model across multiple domains. Worth of note, Garrison et al. (2010) performed a systematic review of CoI model and found a need for further validation across populations and disciplines. The rapid development of online learning, as a result, provides an ideal environment for evaluating the CoI model. Further development of this model, as discussed in the following sessions, is imperative.

### 2.2. A revised CoI model

Acknowledging that the principal argument of this paper is the potential of a further development of the CoI model, it is crucial to highlight the endeavors of Shea and Bidjerano (2010) in expanding the CoI Framework through the incorporation of a new presence, i.e., Learner presence. Their research explored the CoI model and posited that the model could be promoted by adding more fully articulated functions of online students. They further developed the CoI model by adding another presence, known as Learner presence. Be that as it may, in light of Shea and Bidjerano (2010) view, what distinguishes the learner presence is its representation of elements, such as self-efficacy along with other cognitive, behavioral, and motivational constructs that support online learner self-regulations.

**Figure 2:**
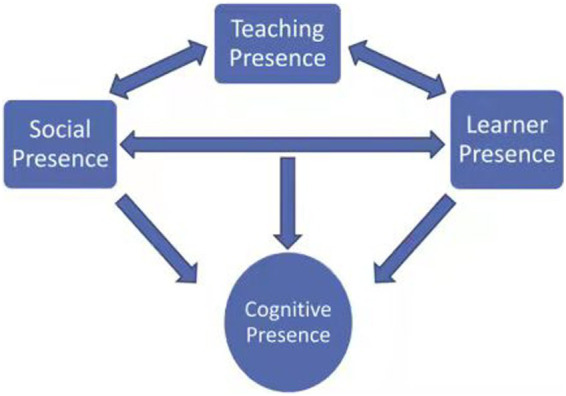
Revised Community of Inquiry model including “Learner Presence.”

Nonetheless, previous research on learner presence has been limited, except for a notable study by Kang et al. (2009), which examined the impact of learner presence on interaction and achievement in web-based project learning. The study demonstrated a significant intersection between learner presence and learning outcomes, including achievement and satisfaction. In a related effort, Shea et al. (2013) extended and confirmed the revised CoI model using quantitative and structural analysis methods (Shea et al., 2013). Additionally, Wertz (2014) evaluated the revised model using confirmatory factor analysis and internal reliability analysis for the four presences, and found that the addition of learner presence improved the CoI model and offered potential for future research. Succinctly, the relationship between the learner presence and the original CoI Model presences is depicted in Figure 2. As shown in Figure 2, learner presence affects cognitive presence and interacts with teaching presence and social presence.

From another perspective, the necessity of incorporating ‘learner presence’ in the questionnaire entailed, as Shea and Bidjerano (2010) carried out, the adoption of the Motivated Strategies for Learning Questionnaire (MSLQ) (Pintrich et al., 1993). Equally worthy as it may, assessing the students’ perceptions of their efficacy and effort demands the implementation of self –efficacy and effort regulation scales from MSLQ. For that reason of necessity, the present paper initially availed itself of the complete seven scales in the MSLQ and subsequently generated five scales following explorative factor analysis.

### 2.3. A model for institution employment of online and blended learning

This paper utilizes the model for institutional employment of online and blended learning in universities developed by Graham et al. (2013) as the theoretical framework for the institutional presence in FDCoI. Basically, the relevant findings of Graham et al. (2013) were composed of three main dimensions: strategy, structure, and support. In this paper, the three scales are integrated into a single scale following explorative factor analysis, and the resulting stages include exploration, adoption, and growth, which illustrate how institutions apply online and blended learning to enhance contribution. Therefore, we suggest that institutional presence represents institutional strategies, structures, and support systems, which facilitate the exploration and implementation of online and blended learning.

Although institutional presence is not directly associated with the learning process, it is inseparable from the process and can, therefore, be considered a component of the CoI model. In view of Crossan et al. (1999), personal and collaborative learning become institutionalized for the purpose of more systematic exploration of learning. It is necessary to consider the institutional factors for learning process because it is helpful for personal and collaborative learning (Crossan et al., 1999). Therefore, the addition of institutional presence into CoI model is both necessary and beneficial. Parker (2008) also emphasized the integration of the teaching and learning procedures in the institutions, the pertinent obligation which lies in expressing sustained commitment to the support of distance learners (Parker, 2008). Given that the administrative function institutions in online and blended learning is essential for students’ satisfaction and learning design (Moisey and Hughes, 2008), institutional presence should be integrated into the CoI model in theory.

### 2.4. Relationship between institutional presence and the other presences

In light of previous research and the associated findings, there is high likelihood that institutional presence dwells in a close connection with teaching and learner presences. Hence, of paramount value to this study is the obvious support which teaching and learner presences benefit from institutional presence in several dimensions. Institutional presence offers support to teaching presence and learner presence in several ways. First and foremost, institutions provide instructional guidance and learning environments that cater to the teaching and learning process. Moreover, instruction criterias of institutions are employed for learning, offering students and teachers with flexible choices for their learning process (Thiessen and Ambrock, 2008). Second, institutions provide policies and regulations governing teaching and learning, including technological support for these processes (Parker, 2008). Administrative support from the institution for students is also critical, as students and institutions mutually benefit from institutional support (Kondra et al., 2008). Third, institutions provide assessments of teaching and learning in the sense that the institution and its teachers afford important evaluation functions that deal with the assessment of student learning (Anderson, 2008). Fourth, institutions support the teaching-learning process by providing students with improved service levels, such as timely academic assistance, significantly enhancing completion rates and student retention, which tangibly benefit both students and institution.

In turn, teaching presence and learner presence possess the potential to positively impact institutional presence and, whilst the institution equally benefits by means of further proactively managing the student relationships and reassuring that learning requirements are met in a timely manner (Kondra et al., 2008). As is shown in the research of Howell et al. (2012), students’ satisfaction is vital for successful online learning, therefore, it is indispensible that institutions and teachers fulfill students’ requirements to provide a satisfactory learning environment.

Consequently, enhancing interaction and reciprocity among institutional presence and learning and teaching presences, it is quite recommendable that learners are aware of services they can expect to receive from the institution and the manner they will be provided (Moisey and Hughes, 2008). On that premise, it is incumbent upon institutions to maintain ongoing communication with students, regardless of their physical location, and ensure that their needs and preferences are duly considered (Shin and Chan, 2004).

Previous studies suggest that institutional presence may closely relate to social and cognitive presence. Social presence is expected to be affected, at least to some extent, by institutional presence, given that institutions invariably govern people’s behaviour and attitudes, and everyone seeks to accommodate each other’s requests, responsibilities, and roles (Davis et al., 2008). Therefore, social presence implies the need for institutional strategies for interaction and supportive policies for building a community. With that in mind, creating an environment where online learning students perceive their institutions and teachers as a model for improving social presence and students’ success (Baker and Edwards, 2011).

Concerning cognitive presence, it is highly probable that links between institutional presence and cognitive presence evenly exist in accordance with Shin and Chan (2004) who proposed that students active in the use of the online learning environment would report a stronger sense of institutional presence compared to the students moderately or weakly interested in gaining information from the online learning environment. Students’ involvement in the online learning environment was, hence, greatly connected to their perceived institutional presence. In other words, the more students engaged with online learning, the stronger their sense of institutional support and connection with their institutions (Shin and Chan, 2004).

### 2.5. Research gap and significance

Based on the preceding literature review, the CoI model has been extensively researched and has matured since its inception as a framework for the three classical presences. However, a research gap remains in the model’s ability to integrate additional presences, such as learner and institutional presence. Although some studies have examined learner presence and its relationship with the original three presences, the gap persists when institutional factors are considered. As institutional factors are inextricably linked to the learning process, adding institutional presence to the CoI model is a promising approach to fill this gap. This paper aims to explore the extent to which institutional presence can be incorporated into the CoI model, given its comprehensive and extensive insights.

This paper’s significance lies in three areas: (1) the CoI model’s historical status and significant role in online and blended learning fields underscores the importance of its development; (2) the addition of institutional presence to the model is a substantial and transformative development, as it alters the model’s structure significantly; and (3) the integration of institutional presence is both necessary and beneficial due to its inseparability from the learning process and its close relationship with the other presences.

## 3. Method

### 3.1. Participants

Data was collected by teachers in five Chinese universities in 2021. Because the goal of the research is to test and discern the relationships among five presences, the primary target was students involved in all the presences. To achieve this, the teachers asked their students to complete the designed questionnaire voluntarily. In total, 762 student participants answered the questionnaire. These participants were all universities students (freshmen, sophomore, and master students) from five Chinese universities (University of Chinese Academy of Sciences, Beijing Institute of Technology, People’s Public Security University of China, Beijing Forest University, and Beijing Union University). The participants came from eleven faculties (Management, Information, Automation, Humanities and Social Sciences, Engineering, Art and Design, Landscape Architecture, The International Institute of Police Law Enforcement, Life Sciences, Resources and Environment, Mechatronic Faculty). Both male and female students were included. Demographic information could be found in Table 1. Although the participants came from different universities and faculties, they were all enrolled in College English and engaged in online and blended learning environments involving individual and group learning. The study design enabled the exploration of the relationships among the five presences within a consistent context.

**Table 1:** Demographic information of research data.

Demographic information (*N* = 762)	
Characteristic	Statistic
*Gender*	
Male	43%
Female	54%
*University*	
University of Chinese Academy of Sciences	5%
Beijing Institute of Technology	16%
People’s Public Security University of China	10%
Beijing Forest University	32%
Beijing Union University	36%
*Faculty*	
Management Faculty	24%
Information Faculty	6%
Automation Faculty	9%
Humanities and Social Sciences Faculty	4%
Engineering Faculty	9%
Art and Design Faculty	3%
Landscape Architecture Faculty	14%
The International Institute of Police Law Enforcement Faculty	10%
Life Sciences Faculty	3%
Resources and Environment Faculty	4%
Mechatronic Faculty	7%
*Grade*	
Master students	20%
Freshmen students	72%
Sophomore students	6%

### 3.2. Instrument

Drawing on the aforementioned theoretical frameworks, a survey instrument comprising five dimensions - Social Presence, Cognitive Presence, Teaching Presence, Learner Presence, and Institutional Presence - was designed. The CoI instrument (Arbaugh et al., 2008) was utilized for measuring the social presence, cognitive presence, and teaching presence, while the Motivated Strategies for Learning Questionnaire (Pintrich et al., 1993) was employed to measure learner presence. Ultimately, the institutional self-evaluation checklist instrument, based on the online and blended learning adoption framework, was used to measure institutional presence.

From this perspective, it is elucidating to accentuate that based on a CoI survey conducted by Garrison and Vaughan (2011), three of the aforementioned dimensions of a learning environments, teaching presence, social presence, and cognitive presence were evaluated (Garrison and Vaughan 2011). In line with the objective of the present study, the CoI Survey asked questions about three elements of learning communities that have been shown to have high internal consistency estimates of reliability: social (*α* = 0.91), cognitive (*α* = 0.95), and teaching presence (*α* = 0.94) (Arbaugh et al., 2008). Out of its intent to elaborate contextually on the CoI survey, this study took the initiative to further develop the said survey by means of an addendum of locally contextualized learner presence and institutional presence to the questionnaire and accordingly testing their relationships. In this questionnaire, there are several sub-dimensions, which are outlined in Table 2. Evidently, each dimension was assigned 3 questions the least. By this pattern, the total questionnaire contained 73 questions (teaching presence 13 questions, social presence 9 questions, cognitive presence 12 questions, learner presence 27 questions, and institutional presence 12 questions). Besides, the questionnaire used a 1–5 Likert scale (1 = strongly disagree, 2 = disagree, 3 = neutral, 4 = agree, 5 = strongly agree). All considered, the total of 762 questionnaire responses were collected, and duly, data was analysed by means of SPSS and AMOS.

**Table 2:** Dimensions and sub-dimensions of the questionnaire.

Dimensions	Sub-dimensions	Sub-dimensions after EFA and CFA
Teaching presence (TP)	Design and organization Facilitation Direct instruction	Design and organization (TPDO) Direct instruction (TPDI)
Social presence (SP)	Affective expression Open communication Group cohesion	Affective expression (SPAE) Open communication (SPOC)
Cognitive presence (CP)	Triggering event Exploration Integration Resolution	Exploration (CPE) Resolution (CPR)
Learner presence (LP)	Intrinsic motivation Extrinsic motivation Self-efficacy Effort regulation Peer learning Time management Student performance	Intrinsic motivation (LPIM) Extrinsic motivation (LPEM) Self-efficacy (LPSE) Peer learning (LPPL) Time management (LPTM)
Institutional presence (IP)	Strategy support	Support (IPSU)

## 4. Results

In this section, the results of descriptive statistics, a normal test, explorative and confirmative factor analysis, and a structural equation model are introduced and explained successively.

The descriptive statistics and Kolmogorov–Smirnov test were conducted with the research variables and questions in the questionnaire. The results, seen in Table 3, show that for the Kolmogorov–Smirnov test, all variables are significant (*p* < 0.05), informing that the data is not normally distributed.

**Table 3:** Results of descriptive statistic and normal test.

	*N*	*M*	SD	*Z*	*P*
LP	762	3.403	0.534	1.780	0.004
LPSE	762	3.529	0.733	2.289	0.000
LPEM	762	3.527	0.793	3.068	0.000
LPIM	762	3.540	0.769	2.416	0.000
LPPL	762	3.249	0.770	3.536	0.000
LPTM	762	3.362	0.572	3.072	0.000
SP	762	3.521	0.678	1.792	0.003
SPOC	762	3.431	0.849	2.714	0.000
SPAE	762	3.567	0.743	3.316	0.000
CP	762	3.564	0.666	1.954	0.001
CPE	762	3.493	0.759	3.252	0.000
CPR	762	3.484	0.786	3.119	0.000
TP	762	3.816	0.706	2.247	0.000
TPDO	762	3.811	0.790	4.245	0.000
TPDI	762	3.811	0.789	4.386	0.000
IP	762	3.415	0.847	2.101	0.000
IPSU	762	3.415	0.847	2.101	0.000

Explorative factor analysis was conducted for five presences separately to reveal the underlying structure of the study’s relatively substantial variables. The results of the explorative factor analysis are displayed in Table 4.

**Table 4:** Main results of explorative factor analysis.

Parameters	Estimated value (CP)	Estimated value (IP)	Estimated value (LP)	Estimated value (SP)	Estimated value (TP)
KMO	0.923	0.958	0.917	0.877	0.954
Bartlett’s test of sphericity (*p* value)	0.001	0.000	0.000	0.000	0.000
Factor 1 eigenvalues	6.424	8.142	8.600	4.646	8.048
Factor 2 eigenvalues	1.159		2.676	1.286	1.088
Factor 3 eigenvalues			1.727		
Factor 4 eigenvalues			1.446		
Factor 5 eigenvalues			1.281		
Cumulative total variance	63.190%	67.852%	58.256%	65.903%	70.275%

Table 4 presents a comprehensive analysis of the data collected, and the results of the explorative factor analysis indicate that the samples for all five presences are appropriate for factor analysis, as demonstrated by the Kaiser-Meyer-Olkin (KMO) measure of sampling adequacy being more significant than 0.7 (*p* < 0.001). The cognitive presence dimension yielded two factors with eigenvalues greater than 1, explaining 63.19% of the variance. The institutional presence dimension delivered a single factor with an eigenvalue greater than 1, explaining 67.852% of the variance. The learner presence dimension yielded five factors with eigenvalues greater than 1, explaining 58.256% of the variance. The social presence dimension also yielded two factors with eigenvalues greater than 1, explaining 65.903% of the variance. Finally, the teaching presence dimension yielded two factors with eigenvalues greater than 1, explaining 70.275% of the variance. The rotated factor matrix shows that each question has a high loading for only one factor, and the factor loading values exceed 0.5, indicating that the factor structures are sound. The validity of the scales is acceptable.

Confirmative factor analysis was then operated to test structural validity further. Eventually, as Table 5 below charts, the results of confirmatory factor analysis can be perceived as follows:

**Table 5:** Main results of confirmatory factor analysis (Bootstrap = 2000).

Model	*χ*2	df	*χ*2/df	TLI	CFI	RMSEA
CP	234.333	51	4.595	0.954	0.964	0.069
IP	255.116	45	5.669	0.961	0.974	0.078
LP	1424.033	313	4.550	0.858	0.874	0.068
SP	128.797	24	5.367	0.954	0.969	0.076
TP	344.190	63	5.461	0.953	0.962	0.077

Because the data is not normally distributed, the Bootstrap method (2000 times) was used to perform parameter estimation. The model fit index after model correction was obtained. As noticeable in Table 5, the main fit index meets the fit requirements, showing the models are acceptable. The factor loadings in the model are higher than 0.5, which indicates that the results of confirmatory factor analysis, the factors’ structure, and scale structure validity are all acceptable.

**Figure 3:**
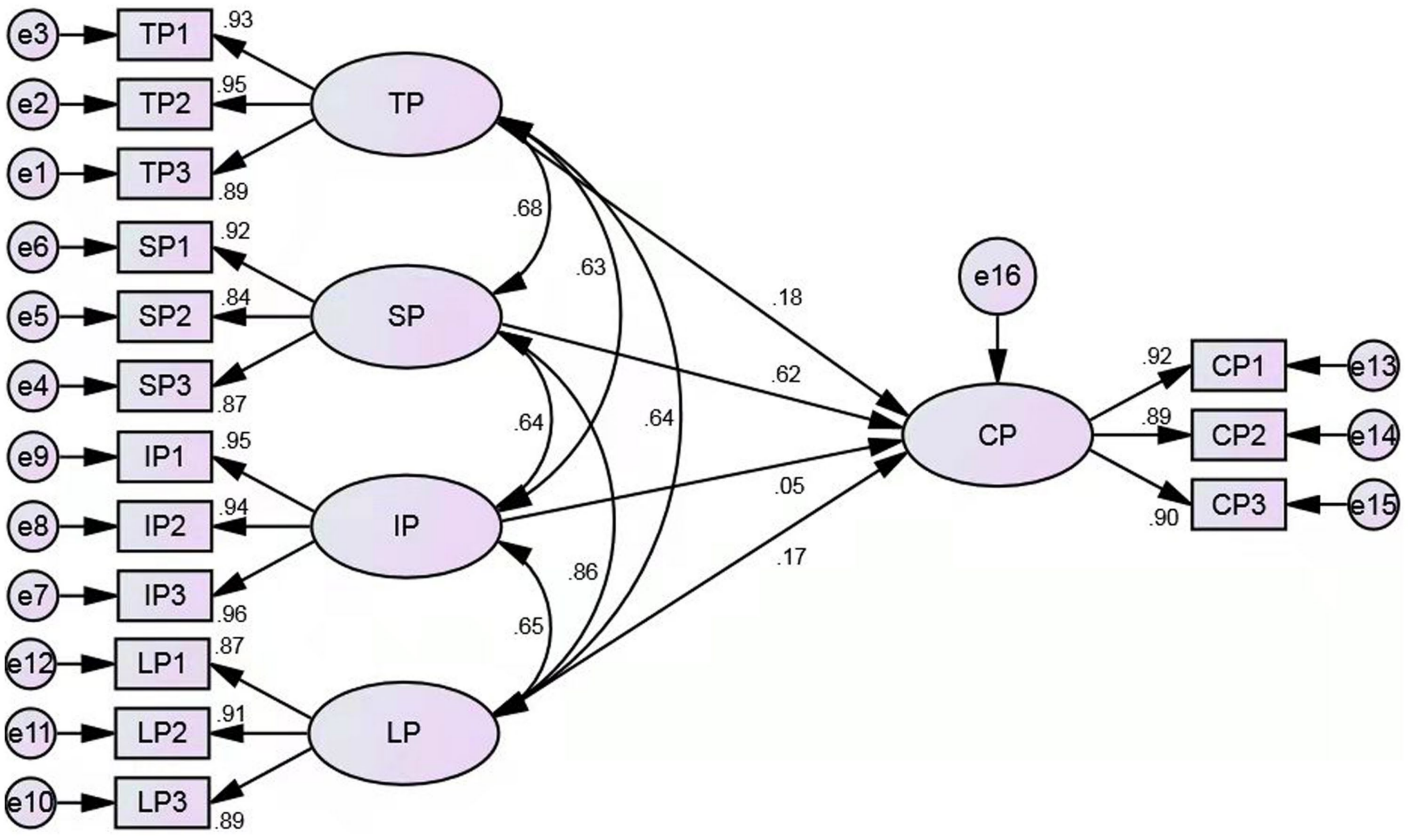
Structural equation model.

Finally, the SEM was conducted to test the relationship among TP, IP, LP, SP and CP. Because the data are a non-normal distribution, the bootstrap method (2000 times) was used to extract the parameters. Regarding the model construction, the latent variables TP, IP, LP, and SP were treated as independent variables, and the latent variable CP was treated as the dependent variable to explore the effects of the independent variables on the dependent variable and the relationships among the independent variables. By virtue of the excessive questions which the questionnaire incorporated, the balance method for packaging was alternatively implemented to simplify the model. That being the case, the questions in each scale were sorted into three packages according to their factor loadings. Finally, the model was generated as schematized in Figure 3. The results meet the applicable requirements, indicating that the model is acceptable and fits well with the data (*χ*2 = 243.165, df = 80, *χ*2/df = 3.040, TLI = 0.984, CFI = 0.987, RMSEA = 0.052). Besides, according to the research of Wertz (2022), the original CoI model with four presences had reasonable model fit [*χ*2 (513) = 900.5, *p* < 0.001; CMIN/DF = 2.25; GFI = 0.83, IFI = 0.92, TLI = 0.91, CFI = 0.92; RMSEA = 0.055)]. Our addition of the institutional presence proves a better optimized CoI model because the CFI value shows a higher degree of the fitting.

The main coefficients in the SEM are demonstrated in Table 6, in light of which the effect of TP on CP was significant (*β* = 0.179, *p* < 0.01). The effect of SP on CP was also significant (*β* = 0.625, *p* < 0.01), but the effect of IP on CP was not significant (*β* = 0.048, *p* > 0.05). The effect of LP on CP was significant (*β* = 0.173, *p* < 0.01). The correlation between TP and LP was significant (*r* = 0.637, *p* < 0.01). The correlation between TP and IP was significant (*r* = 0.63, *p* < 0.01). The correlation between TP and SP was significant (*r* = 0.676, *p* < 0.01). The correlation between SP and IP was significant (*r* = 0.635, *p* < 0.01). The correlation between LP and IP was significant (*r* = 0.651, *p* < 0.01). And the correlation between LP and SP was significant (*r* = 0.858, *p* < 0.01).

**Table 6:** Path coefficients and correlation coefficients in structural equation model (Bootstrap = 2000).

			SE	SE-Bias	Estimate	Lower	Upper	*P*
CP	←	TP	0.037	0.001	0.179	0.108	0.254	0.001
CP	←	SP	0.062	0.001	0.625	0.513	0.748	0.001
CP	←	IP	0.031	0.001	0.048	−0.012	0.109	0.118
CP	←	LP	0.052	0.001	0.173	0.066	0.269	0.002
TP	↔	LP	0.035	0.001	0.637	0.563	0.704	0.001
TP	↔	IP	0.033	0.001	0.63	0.562	0.692	0.001
TP	↔	SP	0.033	0.001	0.676	0.605	0.737	0.001
SP	↔	IP	0.03	0.001	0.635	0.573	0.691	0.001
IP	↔	LP	0.029	0.001	0.651	0.59	0.705	0.001
SP	↔	LP	0.017	0	0.858	0.821	0.889	0.001

## 5. Discussion

If the dominant argument as well as core objective of the present quantitative study lies in its addendum of institutional presence to further develop the CoI Model, the achieved findings reflect the suitability of the model as long as it is relevant to the data. By the same token, institution presence has moderate to strong standardized loadings and significant interactions with teaching presence, social presence and learner presence. Therefore, our analysis of the first research question, which seeks to determine whether institutional presence can be integrated into the CoI model, confirms the positive integration of this construct into the CoI framework. Furthermore, the study’s findings align with Crossan et al.’s (1999) assertion that institutionalized learning is crucial for building on past knowledge and Parker’s (2008) emphasis on the importance of maintaining the integrity of teaching and learning processes within educational institutions. These findings are compelling and intriguing, as they corroborate existing literature and expand our understanding of the CoI model’s potential to analyze institutional presence.

**Figure 4:**
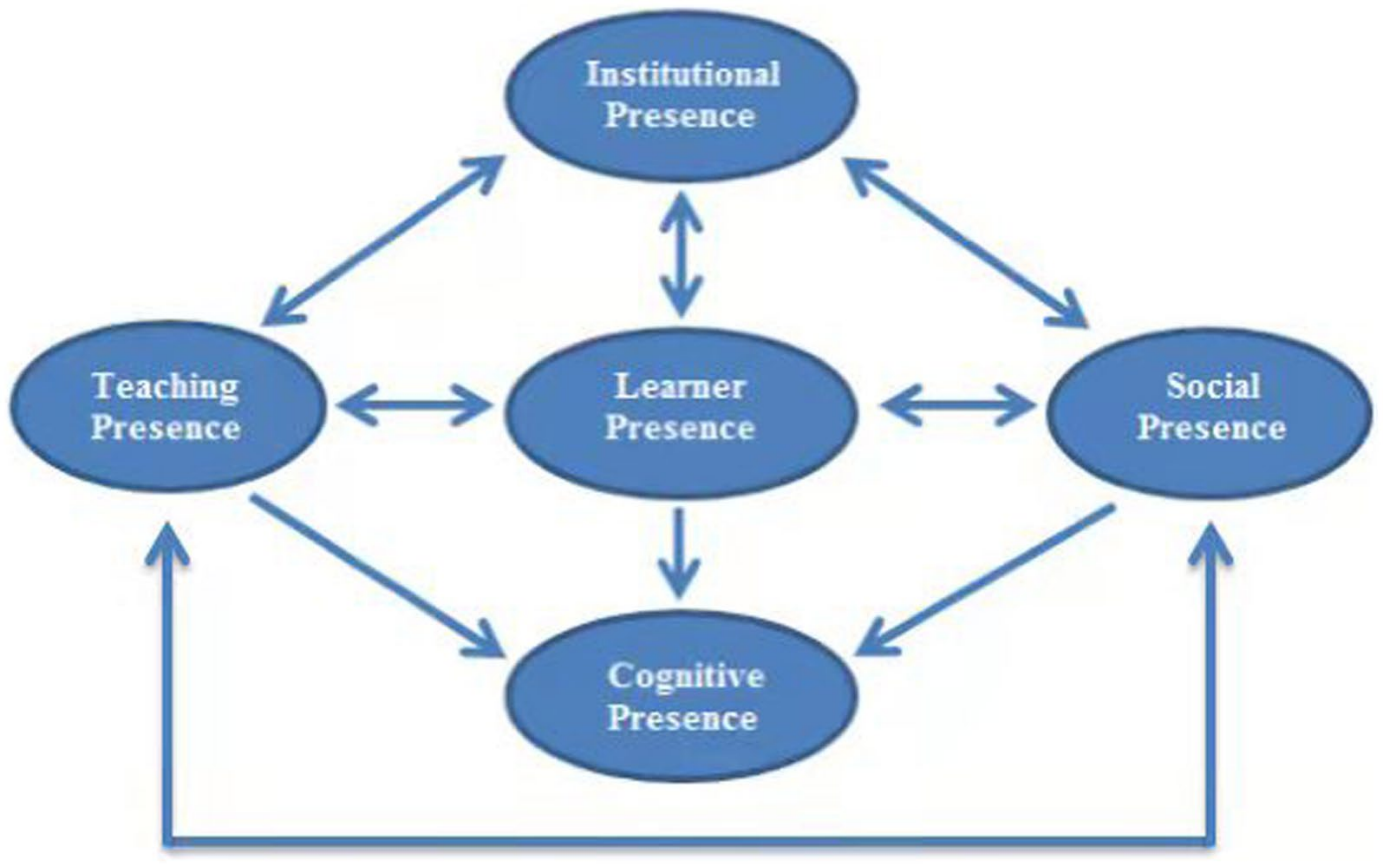
A further developed CoI model including “institutional presence.”

Concerning the second research question (how institutional presence should be integrated into the CoI model?), what ought to be inferred through path analysis and the realized findings is that teaching presence, social presence, and learner presence all significantly affect cognitive presence. In contrast, the effect of IP on CP was not significant (*β* = 0.048, *p* > 0.05). Equally significant, the correlations among IP and other presences (TP, SP, LP) were all significant (*p* < 0.01). On that premise, Figure 4 below is a graphic representation of the further developed FDCoI Model in utter conformity with the achieved results.

The present study examined the relationships among social presence, cognitive presence, teaching presence, and learner presence. The results were consistent with the original CoI model created by Garrison et al. (2000). In terms of the correlation among learner presence and the three presences in the original CoI model, the results of the study were in parallel consistency with the conclusions of Shea and Bidjerano (2010). In terms of institutional presence, while it did not significantly affect cognitive presence, it displayed significant interactions with all other presences (teaching presence, social presence, and learner presence). This suggests that institutional presence is an influential factor and context in the FDCoI model. This finding supports Peacock and Cowan’s (2016) argument that the presences have greater meaning and impact on learning when they are related in strategy to their effects on the learning experience, particularly in remarkable issues in online learning research. However, our research focused on the influential function of the institutional presence and its integration into the CoI model, whereas Peacock and Cowan (2016) explored the interweaving of the presences, explicitly identifying the influence areas as “trusting,” “collaborative learning,” and “deepening understanding.” The perception of institutional presence as an influential factor and context is consistent with Vlachopoulos and Cowan’s (2010) suggestion that context is located outside a “ring fence.” Therefore, it is advisable to incorporate moderated online learning within an enclosed learning arena (ring-fence) that encompasses students’ activities with the e-moderator.

Following confirming the influential context role of institutional presence in the FDCoI model, we further investigated our assumption that the FDCoI is learner-centered. To this end, we intentionally invited students, rather than teachers or institutions, who were involved in all presences, to participate in the study. Given that learners should be inclusively at the center and connected to all the presences in FDCoI, it would be advantageous to grant them a challenging role in creating the learning environment and the social norms for the learning community, including assisting in online team building and ice-breaking activities (Johnson, 2001). Pedagogically worthwhile, this claim is utter congruence with Vlachopoulos and Cowan (2010)’s supposition that online and blended learning is student-centred and implicitly student-directed inputs and instruction do hardly feature within or without the learning arena on the ground that it is necessary to be located beyond the boundaries of this diagram, as sought out by the learners. However, Shea and Bidjerano (2010), who developed the CoI model by adding the learner presence, did not emphasize the student-centred element in their model. Therefore, the highlighting of the learner presence in this study as the center role in the FDCoI model represents a unique contribution to the CoI model. This underscores the importance of considering learners as active participants in the learning process and granting them a significant role in shaping the learning community’s norms and practices.

## 6. Conclusion and limitation

In keeping with its research objective and methodological approach, this paper endeavors to extend the CoI model by incorporating institutional presence through factor analysis and Structural Equation Model. The quantitative findings of this study substantiate the degree to which institutional presence holds the potential to be incorporated in the CoI model by virtue of its excessive and comprehensive insights for proponent researchers and further elaborative investigation. This result accords with one of the hypotheses of this study, that is, the CoI model could be further developed by the addition of an institutional presence. However, the results does not satisfy with another hypothesis of this study, that is, institutional presence possesses significant effect on cognitive presence. According to the results of this study, the effect of institutional presence on cognitive presence was not significant. Nevertheless, as proposed in this paper, the CoI model, with an extension of institutional presence corroborates the parameters of its integration into CoI model and correlation to the other four presences. Besides, the research proves that institutional presence functions as an influential context factor and learner presence serves as the center and connection in the model. The results are in line with the study of Crossan et al. (1999), who argue that institutionalized learning supports and affects personal and collaborative learning. On the basis thereof, the findings can assist other researchers in investigating the systematic cycle of the CoI model and its internal structure. The results further explain and provide insights into the internal structure of the new CoI model and help to demonstrate and verify the new model.

Despite its endeavors to unearth a range of lines of Institutional Presence, this paper reasonably far exonerated from limitations, the most daunting of which resides in its rather confined data domains. By way of a plain and concise explanation, though 762 questionnaires were collected and examined, they hardly sufficed for such a new model to be adequately tested in other domains. Therefore, further research using more extensive data from various countries, universities, knowledge domains, subjects, and grades is necessary. In addition, further research could explore the perceptions of other groups, such as teachers or institutions, to determine whether their views differ from those of students.

To the best of its attempts within the pertinent scope, this paper which has initiatively revisited the CoI model, in a more comprehensive and systematic way, to incorporate ‘institutional presence’ as a value-added element to its pedagogical efficacy. Throughout the various stages of the collected data analysis, institutional presence indicated its firm connection to the other four presences, namely, teaching presence, learner presence, social presence and cognitive presence, and, hence, proved its insightfulness to both instructors and students for advanced exploration.

## Data availability statement

The original contributions presented in the study are included in the article/Supplementary material, further inquiries can be directed to the corresponding author.

## Author contributions

WZ and CZ: conceptualization, methodology, validation, and writing—review and editing. WZ: software, formal analysis, investigation, resources, data curation, writing—original draft preparation, visualization, project administration, and funding acquisition. CZ: supervision. All authors have read and agreed to the published version of the manuscript.

## Funding

This paper was financed by the Humanities and Social Sciences Foundation Project of the Ministry of Education of the People’s Republic of China “Research on the Reconstruction and Practice of Community of Inquiry Model in Blended Learning” (20YJC880123); Beijing Social Sciences Planning Project of Beijing Municipal Education Commission “Comparative Study on the Effectiveness of Online Learning and Traditional Learning in Municipal Universities” (SM202110005009); The Decision Consultation Project of the Beijing Social Sciences Foundation “Evaluation and Countermeasure Research for the Effectiveness of Engineering Blended Learning in Municipal Universities” (22JCC120).

## Conflict of interest

The authors declare that the research was conducted in the absence of any commercial or financial relationships that could be construed as a potential conflict of interest.

## Publisher’s note

All claims expressed in this article are solely those of the authors and do not necessarily represent those of their affiliated organizations, or those of the publisher, the editors and the reviewers. Any product that may be evaluated in this article, or claim that may be made by its manufacturer, is not guaranteed or endorsed by the publisher.
