# Corrigendum: Institutional presence: toward a further developed Community of Inquiry model integrating institutional functions in online and blended learning environment

**DOI:** 10.3389/fpsyg.2023.1292252

**Published:** 2023-09-27

**Authors:** Wei Zhang, Chang Zhu

**Affiliations:** ^1^Higher Education Institute, Beijing University of Technology, Beijing, China; ^2^Higher Education Institute, Vrije Universiteit Brussel, Brussels, Belgium

**Keywords:** institutional presence, Community of Inquiry, model, blended learning, online learning

In the published article, there was an error in the Funding statement as published. The Funding statement included funding that should have been excluded, and excluded funding which should have been included. The correct Funding statement appears below.

